# A Small Key for a Heavy Door: Genetic Therapies for the Treatment of Hemoglobinopathies

**DOI:** 10.3389/fgeed.2020.617780

**Published:** 2021-02-04

**Authors:** Hidde A. Zittersteijn, Cornelis L. Harteveld, Stefanie Klaver-Flores, Arjan C. Lankester, Rob C. Hoeben, Frank J. T. Staal, Manuel A. F. V. Gonçalves

**Affiliations:** ^1^Department of Cell and Chemical Biology, Leiden University Medical Center, Leiden, Netherlands; ^2^Department of Human and Clinical Genetics, The Hemoglobinopathies Laboratory, Leiden University Medical Center, Leiden, Netherlands; ^3^Department of Immunology, Leiden University Medical Center, Leiden, Netherlands; ^4^Department of Pediatrics, Stem Cell Transplantation Program, Willem-Alexander Children's Hospital, Leiden University Medical Center, Leiden, Netherlands

**Keywords:** gene therapy, genome editing, hemoglobinopathies, fetal globin induction, thalasseamia, sickle-cell disease (SCD), hemoglobin switch, gamma-globin

## Abstract

Throughout the past decades, the search for a treatment for severe hemoglobinopathies has gained increased interest within the scientific community. The discovery that ɤ-globin expression from intact *HBG* alleles complements defective *HBB* alleles underlying β-thalassemia and sickle cell disease, has provided a promising opening for research directed at relieving ɤ-globin repression mechanisms and, thereby, improve clinical outcomes for patients. Various gene editing strategies aim to reverse the fetal-to-adult hemoglobin switch to up-regulate ɤ-globin expression through disabling either *HBG* repressor genes or repressor binding sites in the *HBG* promoter regions. In addition to these *HBB* mutation-independent strategies involving fetal hemoglobin (HbF) synthesis de-repression, the expanding genome editing toolkit is providing increased accuracy to *HBB* mutation-specific strategies encompassing adult hemoglobin (HbA) restoration for a personalized treatment of hemoglobinopathies. Moreover, besides genome editing, more conventional gene addition strategies continue under investigation to restore HbA expression. Together, this research makes hemoglobinopathies a fertile ground for testing various innovative genetic therapies with high translational potential. Indeed, the progressive understanding of the molecular clockwork underlying the hemoglobin switch together with the ongoing optimization of genome editing tools heightens the prospect for the development of effective and safe treatments for hemoglobinopathies. In this context, clinical genetics plays an equally crucial role by shedding light on the complexity of the disease and the role of ameliorating genetic modifiers. Here, we cover the most recent insights on the molecular mechanisms underlying hemoglobin biology and hemoglobinopathies while providing an overview of state-of-the-art gene editing platforms. Additionally, current genetic therapies under development, are equally discussed.

## Introduction

Hemoglobinopathies are the world's most common group of monogenic disorders with an estimated 7% of the global population carrying these diseases (Piel, 2016). Particularly prevalent in Africa, Asia and the Mediterranean, hemoglobinopathies are presently distributed globally due to increased migration rates (Williams and Weatherall, 2012; Piel, 2016). The clinical presentation of hemoglobinopathies varies from mild to severe, depending on the type of inherited mutation, the zygosity of the mutation and the co-inheritance status of ameliorating genetic modifiers, such as hereditary persistence of fetal hemoglobin (HPFH).

By applying X-ray crystallography to hemoglobin, Max Perutz pioneered the use of structural information to uncover how mutations lead to disease at the molecular and atomic levels (Perutz, 1962). These and subsequent fundamental insights on hemoglobin biology formed the basis for the development of conventional treatments sustaining the clinical management of hemoglobinopathies, in particular, blood transfusion, iron chelation and pharmaceutical induction of fetal hemoglobin (HbF) (Kohne, 2011). Yet, for severe clinical cases requiring regular blood transfusions, the only curable option is allogeneic hematopoietic stem cell transplantation (allo-HSCT). New insights in pre-transplant evaluation, donor selection, stem cell source and post-transplant management, together with the implementation of reduced-intensity conditioning (RIC) regimens and pre-transplant immunosuppressive therapy, have all greatly improved treatment outcomes. However, allo-HSCT still suffers from limited donor availability issues as well as morbidity and mortality risks associated with suboptimal donor matching (Angelucci et al., 2014; Baronciani et al., 2016; Zaidman et al., 2016; Anurathapan et al., 2020). Moreover, allo-HSCT protocols for sickle-cell disease (SCD) are more difficult to define than those for β-thalassemia major due to their more complex prognostic disease-severity criteria. This makes it more difficult to optimize allo-HSCT protocols for each patient (Angelucci et al., 2014).

The ongoing development of various candidate genetic therapies raises the possibility that autologous hematopoietic stem cell transplantation (auto-HSCT) will complement, or perhaps even replace, allo-HSCT as a preferable treatment modality. Importantly, the efficient and safe collection of long-term repopulating hematopoietic stem cells (HSCs) from patients with severe hemoglobinopathies is crucial for the success of genetic therapies. The development of plerixafor-based mobilization regimens has resulted in an efficient and safe manner of collecting long-term engrafting HSCs from both SCD and β-thalassemia patients (Yannaki et al., 2013; Baiamonte et al., 2015; Boulad et al., 2018; Esrick et al., 2018; Uchida et al., 2020). Of notice, considering the relative high prevalence of hemoglobinopathies in low- to middle-income regions, spanning over sub-Saharan Africa, the Mediterranean, the Middle East and South-east Asia, bottlenecks for a world-wide implementation of both allo- and auto-HSCT are the costs and requirements for specialized centers. For this reason, considerable efforts continue to be directed toward the improvement of standard and easy-to-implement palliative treatments and diagnostics (Taher et al., 2018; Ikawa et al., 2019; Iolascon et al., 2019).

With regard to candidate curative genetic therapies, one can consider three main aspects driving their progression toward clinical application. Firstly, the fundamental understanding of hemoglobin biology, in which knowledge on genetics, cell biology and human development is combined to obtain a comprehensive understanding of the molecular clockwork underlying hemoglobin-linked phenotypes under homeostatic and disease states. Secondly, clinical genetics play a crucial role in allocating the cause for specific hemoglobinopathies and in identifying any disease modifying genetic traits. Lastly, research on innovative genetic techniques, e.g., lentiviral vector (LV)-mediated gene addition, or gene editing based on programmable nucleases, e.g., zinc-finger nucleases (ZFNs), transcription activator-like effector nucleases (TALENs) and RNA-guided nucleases (RGNs) based on clustered regularly interspaced short palindromic repeats (CRISPR)-associated (Cas) proteins (CRISPR-Cas), is required to permanently rescue pathological phenotypes through the transplantation of genetically modified HSCs.

As mentioned before, knowledge on the molecular, cellular and developmental processes underlying hemoglobinopathies is extensive. As such, it provides a rich foundation on which novel genetic therapy concepts can be built upon and tested. Currently, there are two main categories of genetic therapies being developed for treating hemoglobinopathies, i.e., gene therapy and gene editing involving exogenous gene addition and direct modification of endogenous DNA, respectively. Backed by decades of fundamental and pre-clinical research, gene therapy is the first modality of genetic therapy entering clinical trials targeting diseases of the hematopoietic system (Cavazzana et al., 2019).

In this review, we provide an overview of the current understanding of the molecular mechanisms governing hemoglobin biology in homeostasis and disease states. Subsequently, building on this knowledge, we cover the ongoing efforts aiming at the development of gene-centered treatments for hemoglobinopathies and, in the process, discuss the underlying state-of-the-art genetic technologies.

## The Molecular Hemoglobin Clockwork Underlying Health and Disease

**Figure 1:**
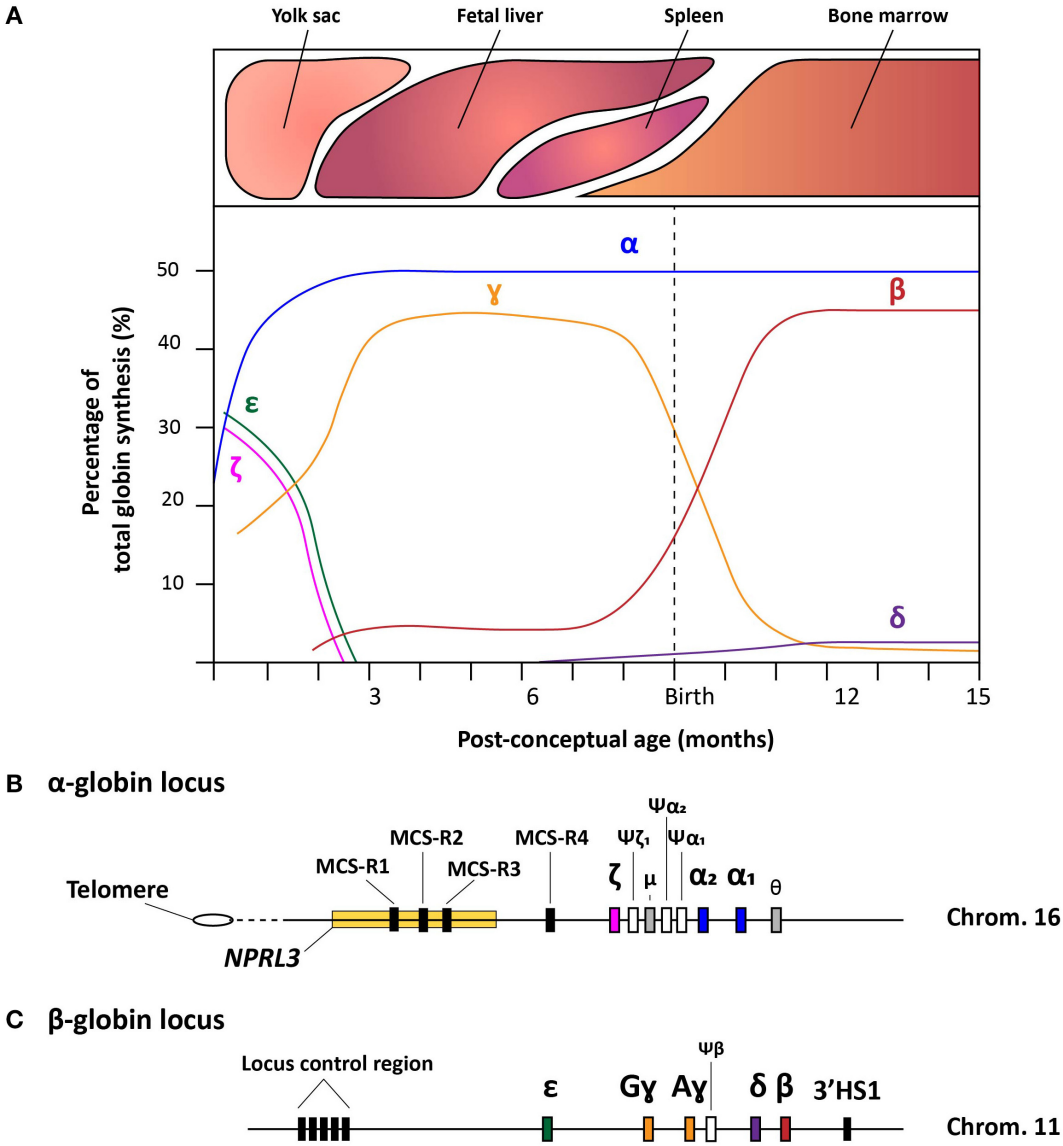
Schematic representation of the hemoglobin switches and the α- and β-globin loci. **(A)** Schematic representation of the human hemoglobin switches before and after birth displaying each globin chain expression levels as the percentage of total globin synthesis. **(B)** Detailed schematic representation of the α-globin locus containing the *NPRL3* gene in which three of the four multispecies conserved regions (MCS-R1-4) lie. The chronological order of ζ-, α_2_-, and α_1_-globin expression follows the genomic order of the respective α-like globin genes, i.e., *HBZ, HBA2*, and *HBA1*, respectively along the cluster. Furthermore, the three inactive globin pseudogenes (Ψ) are depicted together with the theta-gene. **(C)** Schematic representation of the β-globin locus containing the locus control region (LCR), the genes coding functional ε-, Gɤ-, Aɤ-, δ-, and β-globin proteins (i.e., *HBE, HBG2, HBG1, HBD*, and *HBB*, respectively), the β-pseudogene, also known as *HBBP1*, and the 3'HS1 enhancer element.

The fundamentals of hemoglobin biology entail an intricate clockwork of molecular mechanisms that enable a balanced expression of various globin subunits during human development (Cao and Moi, 2002). Hemoglobin is a tetrameric protein present in red blood cells (RBCs) specialized in the transport of oxygen and carbon dioxide throughout the body. It consists of two sets of two identical globin chains, categorized as two α- and two non-α globin chains, each of which forming a heme pocket containing a heme-group with a central iron ion that gives hemoglobin its oxygen carrying capacity and distinctive red color. During human development, the composition of hemoglobin molecules changes in that different pairs of globin subunits assemble in a process called hemoglobin switching. During the first 3 months of gestation, the hemoglobin tetramer starts as embryonic hemoglobin (ζ_2_ε_2_, α_2_ε_2_, and ζ_2_ɤ_2_), then as fetal hemoglobin (α_2_ɤ_2_) and, finally, shortly after birth, acquires the composition of adult hemoglobin (α_2_δ_2_ and α_2_β_2_). These hemoglobin variants are expressed in the embryonic yolk sac, fetal liver, spleen (albeit to a much lower extent compared to the bone marrow) and bone marrow, respectively (Figure 1A). To regulate high expression levels of the various globin genes through the different developmental stages and tissues, besides the canonical *cis-*acting regulatory sequences, a complex, yet robust, regulatory mechanism has evolved (Chada et al., 1985; Townes et al., 1985; Kollias et al., 1986). In the following sections, we provide an overview of the current understanding of this mechanism for both α- and β-like globin expression by discussing the α- and β-globin loci and their regulatory elements (Figures 1B,C).

### Regulation of the α-globin Genes

The ~26 kb α-globin locus contains the ζ-globin gene (ζ or *HBZ*), the duplicated α-globin genes (α_2_ and α_1_, *HBA2* and *HBA1*, respectively), two transcriptionally active genes with unknown function (μ and θ) and 3 pseudogenes (ψζ1, ψα2, and ψα1). This cluster is located near the telomeric end of the short arm of chromosome 16 (Higgs et al., 1989) (Figure 1B) While the ζ- and α-globin genes contribute to the hemoglobin formation during human development, the three pseudogenes are non-functional due to the presence of inactivating mutations. Interestingly, the μ and θ genes show high homology to the α-globin genes and, albeit at much lower rates than ζ, α_2_, and α_1_, they are actively transcribed in erythroid cells, yet, without yielding detectable protein products (Marks et al., 1986; Clegg, 1987; Hsu et al., 1988; Albitar et al., 1992a). It is hypothesized that the μ and θ genes are in transition toward becoming completely inactive pseudogenes, or that the globin-like gene products function sufficiently at very low levels (Goh et al., 2005).

**Figure 2:**
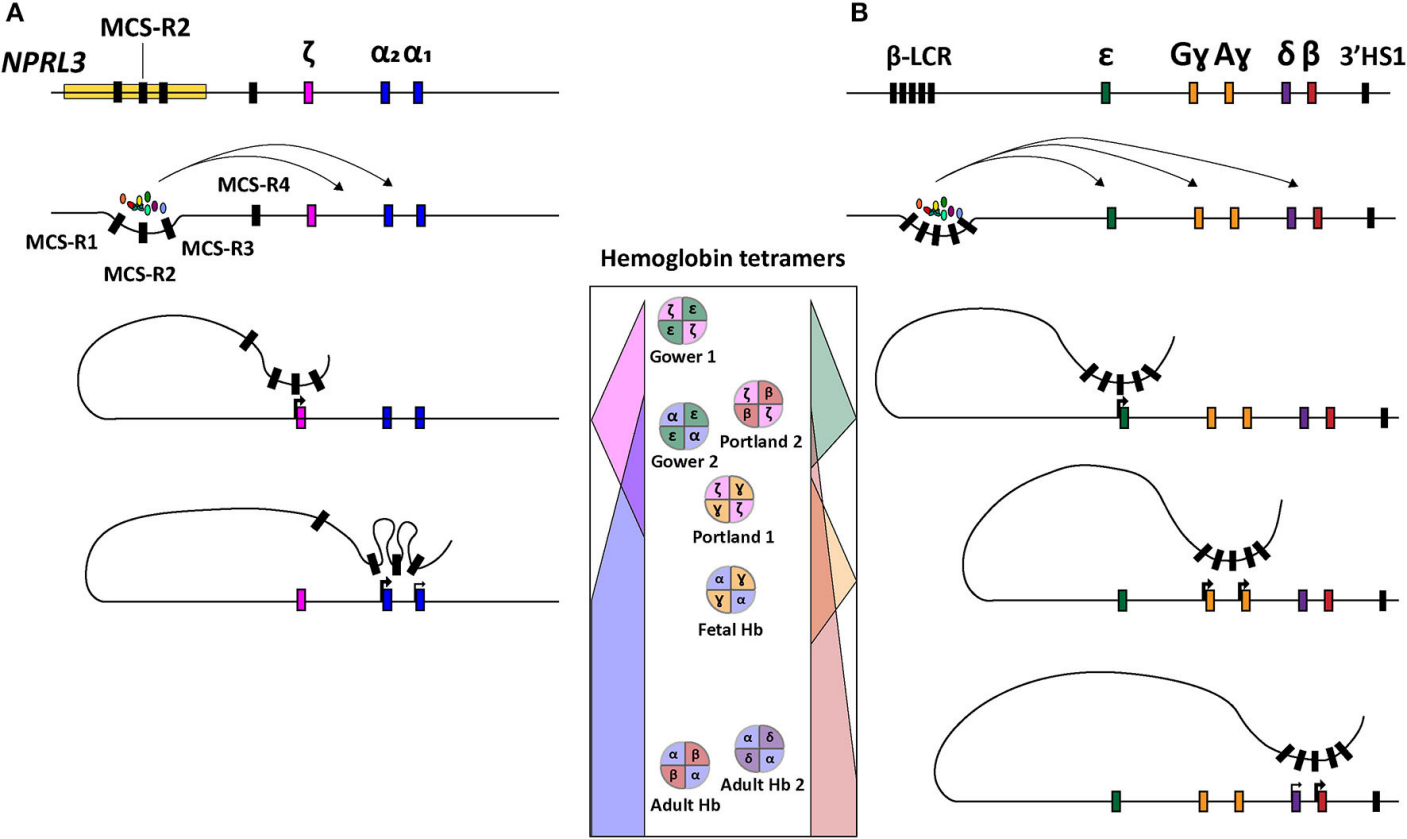
Schematic representation of hemoglobin regulation. **(A)** Structure of the α-globin locus showing the recruitment of tissue-specific transcription factors at the distal regulatory regions (MCS-R1-4) and subsequent sequential chromosomal looping directing the orderly expression of ζ-, α_2_-, and α_1_-globin. **(B)** Structure of the β-globin locus depicting the recruitment of tissue-specific transcription factors at the distal regulatory elements (LCR) and ensuing sequential chromosomal looping governing orderly expression of ε, Gɤ, Aɤ, δ, and β-globin. In the center, the concomittant hemoglobin tetramer variants are displayed according to the chronological order of human development (top-to-bottom).

Concerning the ζ- and α-globin differential expression process, an interesting observation was made through clinical genetics in which a family with α-thalassemia had an intact α-globin locus but carried a large (~62 kb) deletion upstream of the α-globin locus (Hatton et al., 1990; Higgs et al., 1990). In particular, in an ~10–50 kb range upstream of the α-globin cluster four so-called multispecies conserved sequences (MCS-R1-4) were identified as crucial regulatory regions for the expression of the α-globin genes (Hughes et al., 2005) (Figure 1B). From these four MCS regions, MCS-R2, formerly known as DNase I hypersensitivity (HS) site HS-40, is considered the major enhancer since it is the only regulatory element capable of driving α-globin expression by itself (Sharpe et al., 1992; Higgs and Wood, 2008). By using humanized mouse models in which the MCS-R2 element was dissected (Wallace et al., 2007), several studies elucidated key functions of this enhancer in α-globin regulation, i.e., binding of tissue-specific transcription factors, long-range chromatin looping and transcription initiation (De Gobbi et al., 2007; Vernimmen et al., 2009, 2011). More specifically, owing to the presence of multiple conserved binding sites for erythroid-specific transcription factors, such as GATA1, GATA2, NF-E2, and SCL/TAL1 (De Gobbi et al., 2007; Vernimmen et al., 2009), MCS-R2, in concert with the other MCS elements, enhances α-globin gene expression by fostering the recruitment of the transcription preinitiation complex and the formation of long-range intra-chromosomal loops (Vernimmen, 2014) (Figure 2A). Additionally, through the recruitment of JMJD3, the MCS-R2 plays an important role in the eviction of the polycomb repressive complex 2 from the CpG-islands at the α-globin genes, thereby removing the repressive H3K27me3 epigenetic chromatin modification (Garrick et al., 2008; Vernimmen et al., 2011). The importance of the interplay between the α-globin gene cluster and the distal enhancers is also supported by the conservation of the latter elements, spanning an ~135 kb region, in various mammalian species (Tufarelli et al., 2004; Hughes et al., 2005; Philipsen and Hardison, 2018). Interestingly, after the switch from embryonic to fetal hemoglobin, the identical α_1_- and α_2_-globin genes are differentially expressed in that the expression level of the latter is 2-3 fold higher than that of the former (Liebhaber and Kan, 1981; Albitar et al., 1992b). This is presumably due to its positioning closer to the upstream MCS-R1-4 enhancer region (Higgs et al., 1989).

### α-thalassemia

Depending on the number of α-globin genes affected, there are four clinically distinguishable forms of α-thalassemia (MIM # 604131), namely, silent carrier, carrier with symptoms, Hemoglobin H disease and α-thalassemia major. These forms range in severity from no symptomology to a lethal condition named Bart's hydrops fetalis (Farashi and Harteveld, 2018). The causes for α-thalassemia can be found in a variety of mutations that results in compromised α-globin expression and, ultimately, in α- vs. non-α-chain imbalances. The clinical severity of the disease is mainly determined by the number of α-globin genes that are disrupted or deleted. During the adult and fetal developmental stages, the relative excess in β-like globin chains, due to a reduction in α-globin chains, leads to the accumulation of non-functional tetramers called HbH (β_4_) and Hb Bart's (ɤ_4_), respectively. These insoluble tetramers precipitate intracellularly and cause the disruption of the RBC membrane leading to hemolytic anemia. In the case of Bart's hydrops fetalis syndrome, the fetus lacks hemoglobin tetramers containing α-globin altogether making it dependent on the expression of Hb Portland (ζ_2_ɤ_2_) (King and Higgs, 2018) (Figure 2). This temporally delimited gene complementation phenomenon allows the fetus to survive until the 23rd to 38th week of gestation (King and Higgs, 2018).

There are two distinct α-thalassemia-causing genotypes. The first entails the disruption or deletion of one of the two α-globin genes either in one or both alleles and is annotated as α^+^-thalassemia. The second comprises the absence, usually through a large deletion, of both α-genes in *cis*, i.e., on the same chromosomal locus, and is annotated as α^0^-thalassemia. The most frequent type of mutations affecting the expression of the α-globin genes are α^+^-thalassemic deletions of one or both of the α-globin genes (~80%), of which the most common deletions are -α^3.7^ and -α^4.2^ (Farashi and Harteveld, 2018). These small deletions occur through misalignment of the respective Z- and X-homology boxes during meiosis, causing the loss of one of the α-globin genes (Farashi and Harteveld, 2018). The fact that the majority of α-thalassemia-causing mutations are small deletions is attributed to the relatively open chromatin structure at α-globin loci, combined with the high density of homologous sequences in this region in the form of gene duplications and *Alu* repeats (Harteveld et al., 1997; Farashi and Harteveld, 2018). Although less common, a wide range of point mutations causing α^+^-thalassemia have also been documented. These mutations affect a plethora of processes, e.g., α-globin gene transcription, mRNA processing, globin chain stability as well as interactions with α-hemoglobin stabilizing protein (AHSP), internal heme-pocket sites and α-b-globin helix-structures (Farashi and Harteveld, 2018).

An interesting rare syndrome that is associated with α-thalassemia is the X-linked mental retardation syndrome ATR-X (OMIM:301040). Characterized by severe mental retardation and dysmorphic features this syndrome shows striking similarities among patients. The molecular cause of ATR-X are point mutations in the *ATRX* gene (Xq13.3) encoding a chromatin-associated protein belonging to the SNF2 family of helicase/adenosine triphosphatases (Gibbons, 2006). Although the connection between ATRX mutations and α-thalassemia is not completely clear, the ATRX protein was found to be a transcriptional regulator affecting α-globin gene expression (Gibbons et al., 2003, 2008; De La Fuente et al., 2011).

The larger deletions, encompassing both α-globin genes in a single chromosomal locus, causing α^0^-thalassemia, occur less frequently than the smaller deletions found in α^+^-thalassemia. Homozygosity for such an α^0^-deletion causes the development of the aforementioned Hb Bart's hydrops fetalis syndrome. In cases where the ζ-globin gene is also deleted, homozygotes will not survive past the earliest stages of development (Farashi and Harteveld, 2018).

### Regulation of the β-globin Genes

The β-globin locus spans over ~70 kb and expresses five functional globins, in particular, ε, Gɤ, Aɤ, δ, and β from the *HBE, HBG2, HBG1, HBD*, and *HBB* alleles, respectively. The expression from the various β-globin gene cluster members takes place sequentially throughout human development (Cao and Moi, 2002; Stamatoyannopoulos, 2005) (Figures 1A,C and 2B). In addition, the β-globin locus contains a single pseudogene, i.e., *HBBP1* (ψβ), which is inactive (Harris et al., 1984). During the first 6 weeks of gestation, ε-globin is expressed in the embryonic yolk sac and forms embryonic hemoglobin tetramers, i.e., ζ_2_ε_2_ and α_2_ε_2_. Next, ε-globin expression is switched off whilst the expression of the two ɤ-globin genes *HBG2* and *HBG1* starts in the fetal liver forming α_2_ɤ_2_ hemoglobin tetramers. Around birth, during the gradual transition of the main tissue of expression from fetal liver to bone marrow, the synthesis of ɤ-globin in erythroid cells is repressed whilst that of β-globin is activated forming adult α_2_β_2_ hemoglobin tetramers (Figures 1A, 2B). This sequential activation and repression of globin genes, in specific hematopoietic tissues, requires complex mechanisms ensuring proper spatiotemporal control over gene product synthesis. Therefore, similar to the α-globin genes, the expression patterns of β-globin genes is regulated through canonical *cis*-acting elements within or proximal to individual genes that function in concert with a series of distal upstream enhancer elements present at the 5'-end of the locus in a chromosomal segment called the locus control region (LCR) (Crossley and Orkin, 1993; Cao and Moi, 2002).

The functional importance of the LCR was first identified through the observation of β-globin silencing in individuals with deletions in this region causing ɤδβ-thalassemia (Van der Ploeg et al., 1980; Kioussis et al., 1983). The LCR contains four erythroid-specific DNase I HS sites, i.e., HS 1 through 4 (HS1-4), and one constitutive HS site (HS-5) further upstream. Together, these *cis-*acting elements enhance globin gene expression (Cao and Moi, 2002). The enhancer elements HS1-4 contain sequences that interact with various proteins, such as, transcription factors GATA1, TAL1, E2A, LMO2, LDB1 and NF-E2 that, together, cooperate in the recruitment of the RNA polymerase II holoenzyme (Lowrey et al., 1992; Zhou et al., 2004; Liang et al., 2008; Borg et al., 2010; Stadhouders et al., 2014; Cavazzana et al., 2017). Indeed, these DNA-protein interactions facilitate the assembly of structural regulatory conformations, such as loop formation, that ultimately favor transcription initiation (Noordermeer and de Laat, 2008) (Figure 2B). According to their sequential location along the β-globin locus, and hence depending on their relative distance to the LCR, the β-globin genes are differentially regulated during development (Hanscombe et al., 1991).

In the context of recent research aiming at the development of genetic therapies for hemoglobinopathies, a crucial aspect of β-globin gene regulation concerns the fetal-to-adult hemoglobin switch. Owing to the ameliorating effects of HPFH in patients with either β-thalassemia or SCD, there is an increasing number of investigations focused on this particular fetal-to-adult hemoglobin switch. The resulting insights are guiding molecular strategies that aim at relieving the ɤ-globin repressing mechanisms. The most recent insights and therapeutic efforts are discussed later.

### β-thalassemia and Sickle Cell Disease

#### β-thalassemia

β-thalassemia (MIM # 613985) is an autosomal recessive disorder caused by a large spectrum of mutations (>300 known) that reduce or abolish the production of the β-globin chain expressed from the *HBB* gene (Thein, 2013; Kountouris et al., 2014). When a mutation causes a complete or partial reduction of β-globin, it is referred to as a β^0^- or β^+^-thalassemia mutation, respectively. Due to the compromised expression of β-globin, excessive free α-globin chains builds-up intracellularly. This excess forms inclusion bodies that lead to RBC loss due to hemolysis and ineffective erythropoiesis. Hence, the degree to which the β-globin expression is affected by a specific mutation together with the co-inheritance status of genetic modifying traits, such as those conferring elevated ɤ-globin (i.e., HPFH) or reduced α-globin expression, can have a major influence on the clinical presentation of β-thalassemia (Thein, 2018). Additionally, genetic modifiers that ameliorate any secondary complications resulting from the disease pathophysiology (e.g., anemia) or from treatment regimens (e.g., excessive iron loads due to repeated transfusions), are also important parameters determining disease progression and severity (Thein, 2018). Interestingly, in contrast to α-thalassemia, the most common type of mutations in β-thalassemia are non-deletional mutations (Thein, 2013). These non-deletional mutations can affect gene transcription (e.g., promoter disruption), RNA processing (e.g., abnormal splicing due to the creation of cryptic splice sites) and mRNA translation (e.g., generation of premature stop codons) (Thein, 2018). The rarer deletional mutations causing β-thalassemia consist of both small and large deletions encompassing the *HBB* gene itself, the LCR or both (Thein, 2013, 2018).

By virtue of the in-depth knowledge about the complex genetics of β-thalassemia and advanced DNA sequencing technologies, it is currently possible to guide clinical management on the basis of the patient's genotype, i.e., causative mutations and genetic modifiers (Badens et al., 2011; Danjou et al., 2011).

#### Sickle Cell Disease

Similar to β-thalassemia, SCD (a.k.a. sickle cell anemia; MIM #603903) is an autosomal recessive disease affecting normal β-globin function. However, in contrast to β-thalassemia, SCD is caused by a single T → A substitution leading to the translation of valine instead of glutamic acid at position 6 of the β-globin chain (Ingram, 1956; Murayama, 1967). Due to this cell sickling (S) mutation, HbS tetramers carrying the abnormal β^S^ globin chains polymerize through hydrophobic valine interactions that form large HbS polymers (Sundd et al., 2019). As a consequence, RBCs become more rigid and distorted, acquire a sickled shape and suffer from cellular stress, dehydration and hemolysis (Kato et al., 2018; Sundd et al., 2019). The severity of SCD mostly depends on the zygosity underlying the sickle cell trait and on the co-inheritance of other *HBB* mutations, such as, the structural HbC or β-thalassemic mutations (β^+^ or β^0^) (Kato et al., 2018). The most common form of SCD is caused by the Hemoglobin SS genotype (Hb SS) in which a patient inherits HbS alleles from both parents. Together with the co-inheritance of HbS and β^0^ alleles, Hb SS is the most clinically severe form of SCD. Interestingly, co-inheritance of SCD and α-thalassemia occurs frequently, which can have an ameliorating effect on disease severity (Rumaney et al., 2014; Saraf et al., 2014). However, this is not always the case as in these HbS/α-thalassemic patients the occurrence of complications, such as aseptic necrosis and retinal disease, seems to be higher (Saraf et al., 2014). Another important genetic modifier is the co-inheritance of HPFH. In this case, the presence of increased numbers of HbF RBCs (F-cells) dilutes the amount of HbS RBCs thereby reducing their contribution to SCD severity. Moreover, heterologous HbF/HbS tetramers (α_2_β^S^ɤ) do not favor pathologic HbS polymerization (Akinsheye et al., 2011). Interestingly, the most important ameliorating effect of fetal globin on the SCD phenotype is its enhanced oxygen affinity which leads to increased oxygen tension in the RBCs carrying HbS. This prevents sickling as low intracellular oxygen levels are usually required for pathologic HbS polymerization to occur (Henry et al., 2020). Indeed, for instance, polymorphisms in important ɤ-globin-regulating loci (e.g., *BCL11A* and *HBS1L*-*MYB*) leading to ɤ-globin persistence into adulthood can ameliorate the clinical severity of SCD (Lettre et al., 2008; Creary et al., 2009; Makani et al., 2011; Sokolova et al., 2019). For this reason, the investigation of both conventional and genetic approaches that lead to the up-regulation of ɤ-globin synthesis post-birth has acquired particular interest in the search for better SCD treatments.

### The Role of Clinical Genetics and Family Studies

Diagnostics of affected patients as well as asymptomatic carriers of novel genetic variants provide insights into the expression and regulation of the globin genes. A paradigmatic example of this was the discovery in a Dutch family with β^0^-thalassemia that the LCR regulates β-globin expression over a long distance (Van der Ploeg et al., 1980). Indeed, in addition to cell and mouse models, the processes by which distal *cis*-acting elements regulate α- and β-globin gene expression have been investigated extensively through genotyping and phenotyping studies of patients and their families. Sometimes, through these studies, unexpected differences between humans and mice are observed. As mentioned before, the major conserved sequences of the HS-40 region of the α-globin gene cluster consists of four important elements MCS-R1-4 of which MCS-R2 was found to be most essential for α-globin gene expression in mice (Higgs and Wood, 2008). With the introduction of multiplex ligation-dependent probe amplification (MLPA) to screen for copy number variation in the globin gene clusters (Harteveld et al., 2005), deletions and duplications influencing globin expression patterns readily uncovered homozygosity for MCS-R2 deletions in patients suffering from HbH disease (Coelho et al., 2010; Sollaino et al., 2010). This finding suggests a complex role of the MCS-R1-4 elements in α-globin gene regulation.

A major advantage of the hemoglobinopathies as disease models for unraveling gene control processes is the availability of large amounts of diagnostic data from patients and carriers alike. In contrast to many other human recessive diseases, carriers are relatively easy to detect by hematologic and biochemical analyses of their RBCs (Traeger-Synodinos et al., 2015). Moreover, as the globin genes are relatively small they are readily covered by Sanger sequencing, which permits establishing clear genotype-phenotype correlations in an easy and straightforward manner.

Families with unexplained α- or β-thalassemia or with elevated expression of HbF were crucial in the discovery of *trans*-acting factors involved in ɤ-globin gene regulation, such as those encoded by *BCL11A, MYB*, and *KLF1* (Thein, 2018). More recently, for families with unexplained microcytic hypochromic anemia and elevated HbA_2_, or with unexplained β-thalassemia intermedia phenotypes, whole genome sequencing (WGS) analysis revealed the involvement of a hitherto unsuspected trans-acting factor (Spt5) encoded by *SUPT5H*, which, when haplo-insufficient, reduced β-globin gene expression (Achour et al., 2020). Although the exact relationship between SUPT5H and β-globin expression remains to be elucidated, zebrafish studies showed a downregulation of the erythroid transcription factor gata1 as a result of foggy/Spt5 knockdown with a subsequent decrease in embryonic erythropoiesis observed (Taneda et al., 2011). It has been hypothesized that the interaction of foggy/Spt5 with gata1 in zebrafish is comparable to that in humans in which FOG1 and GATA1 cooperate in up-regulating *HBB* expression (Achour et al., 2020). Analyses of these families may provide additional information about how β-globin gene expression is regulated and, in doing so, aid in identifying new targets for treatments based on genetic interventions.

As the field of molecular genetics continues to grow, additional genetic variants in families with rare and unexplained thalassemia phenotypes are expected to be found even before diagnosis are established at the hematological level. For instance, through next generation sequencing (NGS) techniques such as whole exome sequencing (WES) and WGS.

## Genetic Therapies for Hemoglobinopathies

Although the outcomes of allo-HSCT have significantly improved in recent decades, the treatment remains suboptimal due to limited donor availability and associated risks, such as, graft-versus-host disease. Therefore, numerous efforts are being directed to treatments based on auto-HSCT in which the patient's own stem cells are harvested, genetically modified *ex vivo* and reinfused back to the patient. In order to appreciate the full potential of genetic therapies for the treatment of hemoglobinopathies, it is important to understand the wide range of genetic toolsets that are under development. Therefore, in the following sections, we briefly cover the currently available genetic techniques and discuss their testing for the treatment of β-thalassemia and SCD.

### Lentiviral Vector-Mediated Gene Therapy

Initially, realistic prospects for gene therapy of hematological disorders arose with the introduction of ɤ-retroviral vectors (ɤ-RVs), such as those based on the Moloney murine leukemia virus, owing to their ability to stably integrate exogenous DNA into target-cell chromosomes. After the emergence of serious adverse events caused by insertional oncogenesis in a few clinical trials using ɤ-RVs, self-inactivating (SIN) lentiviral vectors (LVs) based on the human immunodeficiency virus type 1 (HIV-1), were introduced (Naldini et al., 1996; Nowrouzi et al., 2011). SIN ɤ-RVs and SIN LVs have viral enhancer sequences present in their long terminal repeats (LTRs) deleted so that, upon chromosomal integration, there is a reduced chance for deregulating cellular genes, e.g., proto-oncogenes (Yu et al., 1986; Zufferey et al., 1998). LVs are especially effective at transducing non-dividing cells as their karyophilic pre-integration complexes do not require mitosis-dependent breakdown of the nuclear envelope to access chromosomal DNA (Naldini et al., 1996). Moreover, LVs have a preference for integrating their reverse transcribed complementary DNA (cDNA) genomes into coding sequences of active genes, whereas ɤ-RVs preferentially integrate near regulatory regions and transcription start sites (Schröder et al., 2002; Wu et al., 2003). Therefore, SIN LVs have become more widely investigated for treating primary immune deficiencies (PIDs), such as, X-linked severe combined immunodeficiency (X-SCID), adenosine deaminase severe combined immunodeficiency (ADA-SCID) and Wiskott-Aldrich syndrome (WAS) (Fischer et al., 2015), as well as for treating metabolic disorders, such as, adrenoleukodystrophy and metachromatic leukodystrophy (Cartier et al., 2009; Biffi et al., 2013).

**Figure 3:**
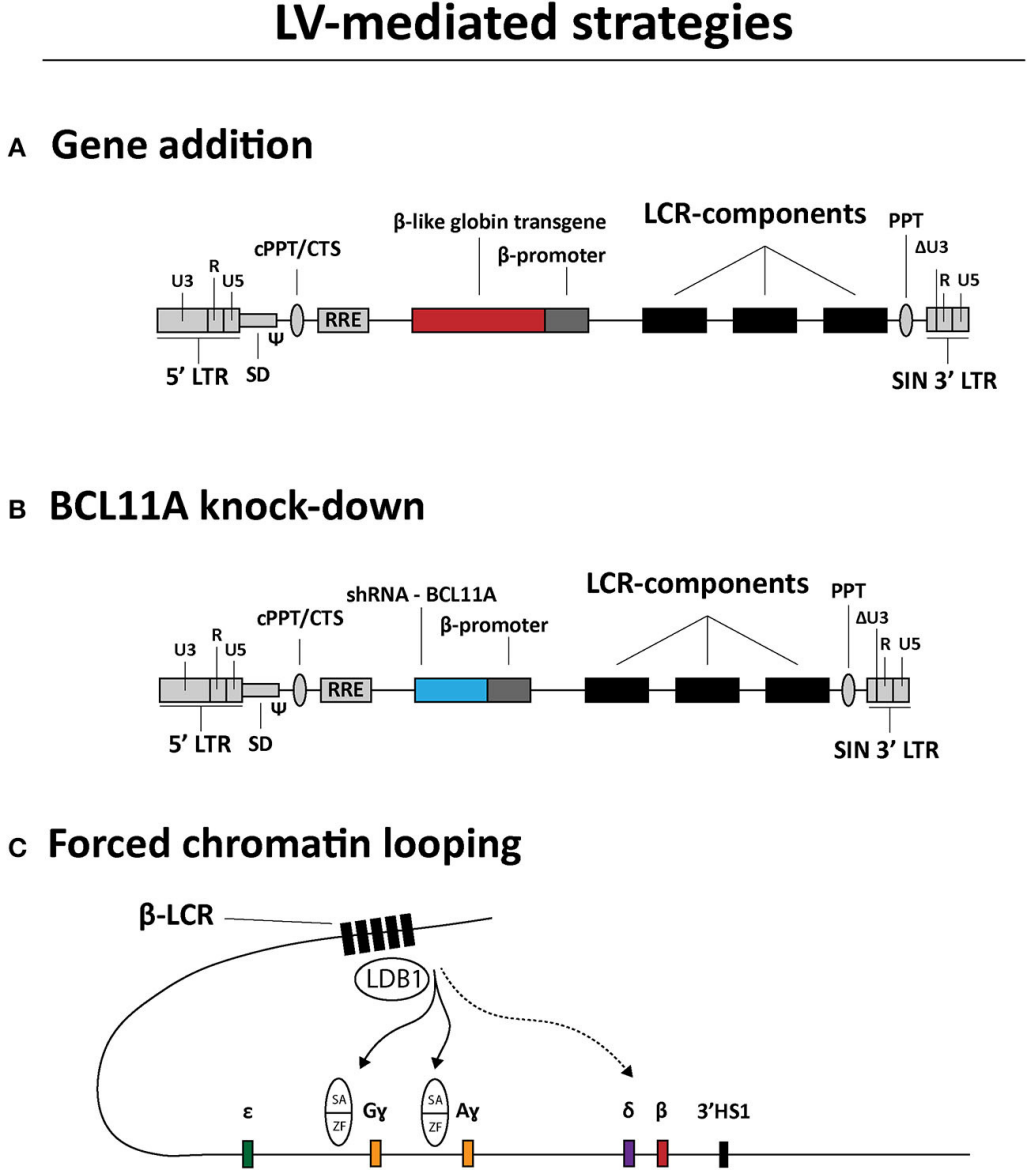
Overview of LV-based gene therapy strategies currently under investigation for the genetic correction of β-hemoglobinopathies. **(A)** Gene addition. Structure of a LV construct for transgene addition relying on the expression of β-like globin chains to rebalance α-chain to non-α-chain ratios. **(B)** BCL11A knock-down. Structure of a LV construct for relieving ɤ-globin repression through shRNA-BCL11A-mediated BCL11A knockdown. **(C)** Forced chromatin looping. Directed loop-formation by LV-mediated delivery of fusion products between LDB1 self-association (SA) domains and zinc-finger (ZF) arrays targeting the *HBG2* and *HBG1* promoter sequences to up-regulate Gɤ- and Aɤ-globin synthesis, respectively. LCR, locus control region; LTR, HIV-1 long terminal repeat; Ψ, HIV-1 packaging signal; RRE, Rev response element; cPPT/CTS, central polypurine tract and central termination sequence; ΔU3, 3' LTR with U3 deletion for self-inactivation of HIV-1 regulatory sequences upon chromosomal integration of recombinant LV genomes.

LVs are currently being tested for treating hemoglobinopathies as well. The main strategies can be categorized in (i) transgene addition (Figure 3A), (ii) short hairpin RNA (shRNA)-mediated *BCL11A* knockdown (Figure 3B) and (iii) forced chromatin looping (Figure 3C) (Breda et al., 2016; Cavazzana et al., 2017; Sii-Felice et al., 2020). The first strategy preceded the other two and is currently the most common and advanced in terms of clinical translation. Indeed, there are a variety of efforts directed at generating LVs carrying recombinant β-like globin gene sequences with the goal of achieving therapeutic levels of transgene expression in RBCs derived from LV-transduced HSCs. To this end, the combined optimization of LCR elements, transgenes and vector genomes is permitting the achievement of efficient transduction of HSCs and subsequent therapeutic protein levels in RBCs (May et al., 2000; Negre et al., 2015; Cavazzana et al., 2017; Sii-Felice et al., 2020). For instance, concerning the optimization of LCR elements in particular, it was demonstrated that incorporating in LV genomes large sequences spanning the HS2, HS3 and HS4 elements, instead of the respective minimal core sequences, yielded sustained and high amounts of recombinant β-globin in RBCs of transplanted mice (May et al., 2000). An initial β-thalassemia gene therapy course was tested in a transfusion-dependent β^E^/β^0^ patient using the Lentiglobin HPV569 vector (Malik et al., 2005; Cavazzana-Calvo et al., 2010) (Table 1). This HPV569 vector carries a cassette encoding a β-globin chain containing an amino acid substitution (β^T87Q^) found in the ɤ-globin chain. Of note, this mutation prevents the polymerization of this recombinant β-globin chain with residual HbS hemoglobin molecules in SCD patients, making the treatment applicable to treat both β-thalassemia and SCD (Adachi et al., 1994; Pawliuk et al., 2001; Negre et al., 2016). Importantly, to achieve high levels of β^T87Q^ globin chain synthesis, the LV genome harbors a minimal β-globin gene promoter together with three LCR elements, i.e., HS2, HS3, and HS4. Finally, upon reverse transcription of the incoming vector RNA genomes and ensuing cDNA chromosomal integration, the β^T87Q^ globin transgene becomes flanked by two copies of the chicken β-globin HS4 chromatin insulator (cHS4) located in the vector LTRs. The enhancer-blocking properties of the cHS4 insulator aims at reducing the chance for insertional oncogenesis due to spurious LCR-driven activation of nearby proto-oncogenes (Emery et al., 2000; Arumugam et al., 2007). Yet, despite achieving high-level βT87Q-globin expression, the HPV569 vector had to be redesigned due to low functional titers and transcriptional activation of the cellular *HMGA2* gene resulting in clonal expansion (Cavazzana-Calvo et al., 2010; Negre et al., 2016). More specifically, chromosomal integration of the vector cDNA in a few cells originated aberrant *HMGA2* transcripts whose origins were mapped to a cryptic splice site in the cHS4 insulator core present in the 5' LTR (Negre et al., 2016). The newly designed vector, named BB305, lacks the cHS4 insulator and contains a hybrid 5'-LTR with cytomegalovirus *immediate-early* gene regulatory elements (CMV) to increase vector RNA synthesis during its production yielding, as a result, higher titers of functional vector particles (Negre et al., 2016).

**Table 1:** Genetic therapies in development based on SIN LVs.

**Phase**	**Product name**	**Clinical trial**	**Vector**	**Therapeutic element**	**Disorder**	**Sponsor**	**Status**	**Results**
I/II	Lentiglobin (HPV569)	LG001	SIN LV	β(T87Q)	TM	Bluebird Bio	Completed	1 patient (β0/βE): 1/1 TI
I	TNS9.3.55	NCT01639690	SIN LV	Wildtype β-globin	TM	Memorial Sloan-Kettering Cancer Center	Active/Not recruiting	Insufficient engraftment and clinical benefit
I/II	Lentiglobin (BB305)	NCT01745120 (HGB-204)	SIN LV	β(T87Q)	TM	Bluebird Bio	Completed	Total: 16/18 reached primary endpoint. Non-β0/β0: 8/10 TI; 2/10 73% and 43% reduction of ATV. β0/β0: 3/8 TI; 1/8 TI for 13 months; 4/8 53% reduction of ATV
I/II	Lentiglobin (BB305)	NCT02151526 (HGB-205)	SIN LV	β(T87Q)	TM & SCD	Bluebird Bio	Completed	TM: 3/4 TI. SCD: three patients had HbAβ(T87Q) contribution of 47.9%, 7.9% and 25.8%
I/II	ZYNTEGLO (Lentiglobin BB305)	NCT02140554 (HGB-206)	SIN LV	β(T87Q)	SCD	Bluebird Bio	Active/Not recruiting	Group A (BMH; *n* = 7): HbAb(T87Q) 0.5-1.2 g/dl. Group B (BMH & MA; *n* = 2): 3.2-7.2 g/dl. Group C (MA; *n* = 32): 22/32 >40% of HbAb(T87Q) contribution (total Hb 9.6-15.1 g/dL; HbAb(T87Q) 2.7-8.9 g/dL). 19/32: Complete elimination of VOCs at 24 months after treatment
III	ZYNTEGLO (Lentiglobin BB305)	NCT02906202 (HGB-207)	SIN LV	β(T87Q)	TM	Bluebird Bio	Active/Not recruiting	HbAβ(T87Q) contribution of: 79.8% (6 months; *n* = 11); 74.2% (12 months; *n* = 8); 77.2% (18 months; *n* = 2)
III	ZYNTEGLO (Lentiglobin BB305)	NCT03207009 (HGB-212)	SIN LV	β(T87Q)	TM	Bluebird Bio	Active/Recruiting	15 TDT patients. 6/8 evaluable patients TI for median 13.6 MPT (Median Hb: 11.5 g/dl). 11/13 patients TI >7 MPT with HbAβ(T87Q) 8.8–14.0 g/dl
Long-term follow-up	ZYNTEGLO (Lentiglobin BB305)	NCT02633943	SIN LV	β(T87Q)	TM & SCD	Bluebird Bio	Enrolling by invitation	32 patients (22 phase I/II; 10 phase III): 14/22 and 9/10 TI (TI patients remained TI for median 39.4 months (min-max:19.4-69.4 months)
I/II	OTL-300 (GLOBE)	NCT02453477	SIN LV	Wildtype β-globin	TM	IRCCS San Raffaele - Telethon	Closed	7 patients (β0 or severe β+). Adults: 3/3 reduced transfusion requirement. Children: 3/4 pediatric patients TI
Long-term follow-up	OTL-300 (GLOBE)	NCT03275051	SIN LV	Wildtype β-globin	TM	Orchard Therapeutics	Active/Not recruiting	8/9 patients (β0 or severe β+) reached primary endpoint after 1 year. Adults: 3/3 reduced transfusion requirement. Children: 4/6 TI; 1/6 reduced transfusion requirement; 1/6 no reduced transfusion requirement due to poor engraftment
I/II	ARU-1801 (RVT-1801)	NCT02186418	SIN LV	ɤ(G16D)	SCD	Aruvant	Active/Recruiting	2 βS/β0 patients with RIC: Excellent safety, feasibility, minimal post-transplant toxicity, and sustained genetically modified cells in PB and BM over 1 year after treatment
I/II	Lenti-βAS3-FB	NCT02247843	SIN LV	β(T87Q/E22A/G16D)	SCD	Donald Kohn (University of California)	Active/Recruiting	No results published yet
I/II	DREPAGLOBE (GLOBE1-βAS3)	NCT03964792	SIN LV	β(T87Q/E22A/G16D)	SCD	Assistance Publique - Hôpitaux de Paris	Active/Recruiting	No results published yet
I	BCH-BB694	NCT03282656	SIN LV	miRNA - BCL11A	SCD	David Williams (Boston Children's Hospital)	Active/Recruiting	6 patients treated. 6/6: Stable HbF induction (20.4-41.3%). Robust increase in F-cells among RBCs (58.9-93.6%).
I	CSL200	NCT04091737	SIN LV	ɤ(G16D) + shRNA734 (BCL11A)	SCD	CSL Behring	Active/Recruiting	No results published yet

*TM, β-thalassemia major; SCD, Sickle cell disease; TI, Transfusion independent; ATV, Annual transfusion volume; BMH, Bone marrow harvest; MA, Mobilization/apheresis; MPT, Months post-treatment; RIC, Reduced-intensity conditioning regimen; VOC, Vaso-occlusive crises; PB, Peripheral blood; BM, bone marrow*.

Since the initial development of the aforementioned Lentiglobin vectors HPV569 and BB305, other LV-based β-thalassemia and SCD gene therapy products have emerged and entered clinical trials, namely GLOBE, OTL-300, ARU-1801, Lenti-βAS3-FB, and DREPAGLOBE (Sii-Felice et al., 2020) (Table 1). The genomes of these LVs differ among each other in several aspects, namely: (i) LCR HS site composition (HS2-4), (ii) type of transgene (i.e., wildtype β-globin, β^T87Q^-globin or hybrid βɤ-globin), (iii) absence or presence of insulators (i.e., cHS4 or FII/BEAD-A; Ramezani et al., 2008); and (iv) vector promoter sequences (i.e., hybrid CMV/5'LTR or wildtype 5'LTR; Cavazzana et al., 2017; Sii-Felice et al., 2020).

Interestingly, intra-femoral injection of HSCs into immunodeficient NOD/SCID recipient mice was shown to increase the frequency of long-term repopulating cells when compared to intravenous injection and, at the same time, reduce the trapping of HSCs in non-target organs (Yahata et al., 2003; Feng et al., 2008). Based on these findings, a clinical trial with the GLOBE vector included the intra-bone infusion of genetically modified HSCs, which, so far, has yielded promising clinical results in terms of rapid HSC engraftment and ensuing hematopoietic system reconstitution (Marktel et al., 2019).

Moreover, a recent study has employed a “forward-oriented” SIN LV design, in which the therapeutic β-globin transgene is transcribed in the “forward” instead of the “reverse” orientation relative to the vector backbone, which is used in the previously mentioned LVs (Uchida et al., 2019). In this specific vector design, the *HBB* intron 2 is not spliced out during vector production owing to the incorporation of the HIV-1 Rev response element (RRE) within this intron. This redesigned vector can be produced at 6-fold higher titers and transduces HSCs at 4- to 10-fold higher rates than their “reverse-oriented” counterparts (Uchida et al., 2019). Importantly, the transduced HSCs showed robust long-term engraftment and β-globin expression in Rhesus macaques up to 3 years post-transplantation (Uchida et al., 2019).

Besides gene addition strategies, a LV expressing a microRNA-adapted shRNA (shRNA^miR^), constructed for inducing fetal ɤ-globin synthesis, has also progressed to a clinical trial stage (Table 1; Brendel et al., 2020). Before its application in a clinical setting, this LV was optimized to minimize the cytotoxicity of shRNA expression and BCL11A knock-down in HSCs (Brendel et al., 2016). In particular, by implementing erythroid lineage-specific expression of the shRNA^miR^ directed to *BCL11A* transcripts, a 90% reduction of BCL11A protein levels led to a 60–70% increase in ɤ-globin expression. Importantly, the genetically modified HSCs were capable of long-term engraftment in mice (Brendel et al., 2016). This optimized LV vector, named BCH-BB694, achieved efficient transduction of healthy and SCD CD34^+^ donor cells as determined by quantification of vector copy numbers (VCNs) resulting in a 3- to 5-fold increase in HbF amounts when compared to mock-transduced cells (Brendel et al., 2020). The demonstration of high CD34^+^ cell transduction efficiencies, without compromising HSC function, together with the ability to produce the BCH-BB694 vector at high titers under good manufacturing practices (GMP) conditions, has permitted the initiation of a phase 1/2 clinical trial (Table 1).

Another LV-based experimental gene therapy aiming at enhancing ɤ-globin expression involves forcing the looping of the LCR toward the ɤ-globin promoter region (Deng et al., 2012, 2014; Breda et al., 2016; Krivega and Dean, 2016) (Figure 3C). In this strategy, the regular role of the transcription factor LDB1 in loop-formation is exploited by expressing a fusion product consisting of the LDB1 Self-Association (SA) domain linked to zinc-finger motifs that recognize the β-globin gene promoter (Deng et al., 2012). After the demonstration of the effectiveness of this elegant forced-looping approach bringing the LCR in close vicinity of the β-globin gene promoter in GATA1 null erythroblasts, a similar strategy was tested for inducing ɤ-globin expression in primary human adult erythroblasts. This resulted in an impressive 85% increase in LCR/ɤ-globin gene contacts and a corresponding up-regulation of ɤ-globin expression (Deng et al., 2014).

### Genome Editing: Basic Principles and Platforms

In this section we focus on gene editing applications for the treatment of hemoglobinopathies. A brief summary is provided on common gene editing platforms, including ZFNs, TALENs and RGNs, as well as on recent gene editing approaches comprising RGNs with high-fidelity Cas9 variants and Cas9 nickase-based techniques, such as base editing and prime editing.

Programmable nucleases can modify specific genomic sequences in eukaryotic cells in a highly precise and efficient manner to, for instance, study the function of genes or correct genes associated with human disorders, both acquired and inborn (Zittersteijn et al., 2020). With the increasing number of candidate genetic therapies entering clinical trials such as those discussed in the previous section, it is clear that the application of advanced therapy medicinal products (ATMPs) is gaining momentum (Hirakawa et al., 2020). Interestingly, feeding the progression of safer and more targeted ATMPs, innovative genome editing techniques are under development, including those directed at treating hemoglobinopathies (Cornu et al., 2017; Li H. et al., 2020).

**Figure 4:**
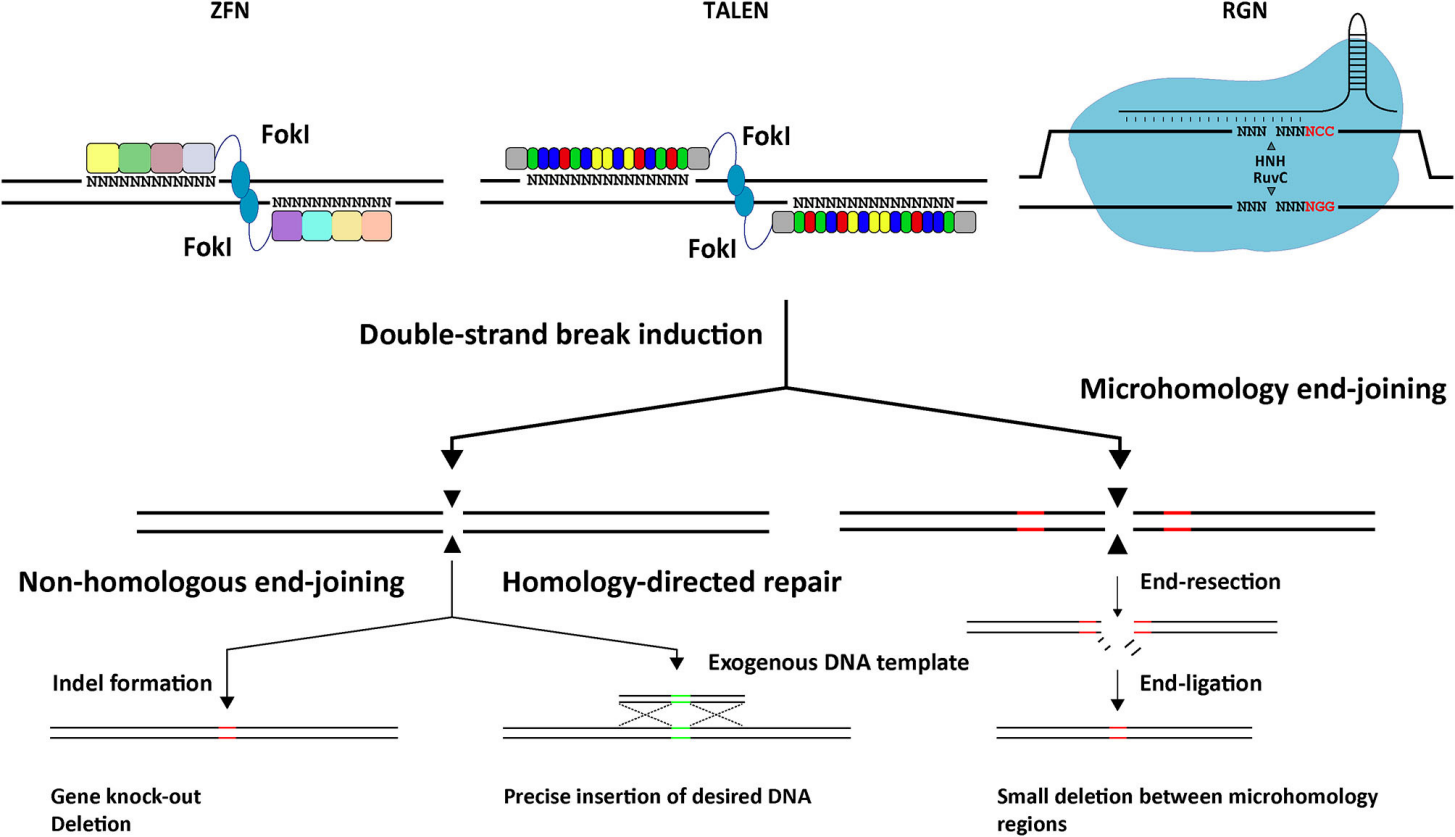
Schematic representation of the three main classes of programmable nucleases (i.e., ZFNs, TALENs, and RGNs based on the prototypic *S. pyogenes* CRISPR-Cas9 system) and the DNA repair pathways underlying different gene editing outcomes resulting from the induction of targeted double-strand breaks. See text for details.

Typically, programmable nuclease-assisted gene editing consists in inducing targeted chromosomal DSBs in living cells to trigger endogenous DNA repair pathways in bringing about specific genetic changes. There are two main processes by which a DSB can be repaired in mammalian cells, namely, (i) end-to-end ligation of chromosomal termini, involving non-homologous end joining (NHEJ) pathways, e.g., classical NHEJ and microhomology-mediated end joining (MMEJ), and (ii) exogenous (donor) DNA-templated repair, involving homology-directed repair (HDR) (Chandrasegaran and Carroll, 2016; Chang H. H. Y. et al., 2017; Figure 4). By building on this knowledge, programmable nucleases are designed to target specific genomic sequences and establish desired gene editing outcomes (e.g., gene knock-ins or knock-outs) (Maggio and Gonçalves, 2015). In mammalian cells, the most active DNA repair pathway is the classic NHEJ which usually results in the precise re-ligation of the chromosomal ends. However, multiple cycles of cleavage and re-ligation caused by the presence of a programmable nuclease, can eventually lead to small insertions or deletions (indels) that, by disrupting the nuclease recognition sequence, become permanently installed in the target cell population (Chandrasegaran and Carroll, 2016; Chang H. H. Y. et al., 2017) (Figure 4). When established at gene coding sequences, these indels lead to frameshifts that effectively result in target gene knock-outs (Chandrasegaran and Carroll, 2016). NHEJ and MMEJ can also be exploited to knock-in exogenous donor DNA into a programable nuclease target site. Often, however, the resulting junctions between donor and target DNA harbor indels. More predictable and precise DNA edits are accomplished via HDR after introducing into target cells an exogenous donor DNA template containing sequences homologous to the target site region together with a cognate programmable nuclease (Chandrasegaran and Carroll, 2016). HDR-mediated gene editing is, however, restricted to the S and late G2 phases of the cell cycle and, as a consequence, has its utility limited to dividing cells.

### Genome Editing Platforms

Clearly, genome editing based on ZFNs, TALENs and RGNs allows for a more precise genetic engineering of cells and organisms than that offered by retroviral vector systems, which suffer from heterogeneous transgene expression levels and insertional mutagenesis risks inherent to their semi-random integrative nature (Schröder et al., 2002; Wu et al., 2003; Maldarelli et al., 2014). Programmable nucleases should, ideally, act in a temporally limited “hit-and-run” fashion, especially when applied in a translational setting to minimizing off-target effects. Regardless, these gene editing tools bear the risk of inducing unwanted genome-modifying events, in the form of off-target indels, chromosomal translocations and, most pervasively, on-target indels and large rearrangements (Cradick et al., 2013; Fu et al., 2013; Hsu et al., 2013; Zhang et al., 2015; Kosicki et al., 2018; Carroll, 2019; Chen et al., 2020). To monitor off-target effects in particular and assess their potential risks, an increasing number of genome-wide screening methods is building up (Zischewski et al., 2017; Kim et al., 2019), and include, GUIDE-seq (Tsai et al., 2015), LAM-HTGTS (Frock et al., 2015; Chen et al., 2020) and, more recently, DISCOVER-seq (Wienert et al., 2019). Insights from applying these methodologies are guiding the optimization of genome editing tools, focusing on improving their specificity without compromising their targeted DSB formation efficiencies.

#### Zinc-Finger Nucleases

ZFNs were the first broadly used programmable nuclease platform (Kim et al., 1996; Chandrasegaran and Carroll, 2016) (Figure 4). These artificial modular proteins consist of an array of Cys_2_His_2_ zinc-finger motifs (typically 4 to 6), each designed to recognize a DNA triplet, fused to a catalytic domain derived from the FokI restriction enzyme. Each ZFN monomer recognizes a specific sequence of 12 to 18 nucleotides with the induction of a DSB requiring the dimerization of two FokI catalytic domains brought together by target DNA binding of a working pair of ZFN monomers (Rahman et al., 2011; Chandrasegaran and Carroll, 2016). The assembly of functional ZFNs with high specificity is complicated due to context-dependent effects. In particular, the fact that individual zinc-fingers can alter the orientation of adjacent motifs or interact with triplets recognized by neighboring motifs (Rahman et al., 2011; Chandrasegaran and Carroll, 2016).

#### Transcription Activator-Like Effector Nucleases

TALENs were developed on the basis of the discovery that TALE proteins found in certain phytopathogenic bacteria (e.g., *Xanthomonas* sp.), are capable of recognizing specific DNA sequences through their DNA-binding units called TALE repeats (Christian et al., 2010; Miller et al., 2011; Chandrasegaran and Carroll, 2016) (Figure 4). In particular, the finding that individual TALE repeats bind via their, so-called, repeat variable di-residues (RVDs) to specific nucleotides on the DNA (Boch et al., 2009; Moscou and Bogdanove, 2009). Hence, customizing TALE DNA-binding domains to a predefined target sequence simply requires the assembly of an array of TALE repeats in which each repeat is predicted to interact with its cognate nucleotide. Similarly to ZFNs, TALENs are artificial modular proteins consisting of a DNA-binding domain fused to the FokI nuclease domain that, through target DNA binding of a working TALEN pair, dimerizes and induces a site-specific DSB (Christian et al., 2010; Miller et al., 2011; Chandrasegaran and Carroll, 2016). Equally similar to ZFNs, once optimized, TALENs exhibit high specificities and efficiencies. Yet, the process of producing and validating TALENs is typically less complex and laborious than that of ZFNs as, in contrast to the binding of individual zinc-finger motifs to target DNA triplets, the binding of TALE repeats to their target nucleotides is substantially less altered by the type of context-dependent effects that zinc-fingers suffer from (Mussolino and Cathomen, 2012).

#### RNA-Guided Nucleases

**Figure 5:**
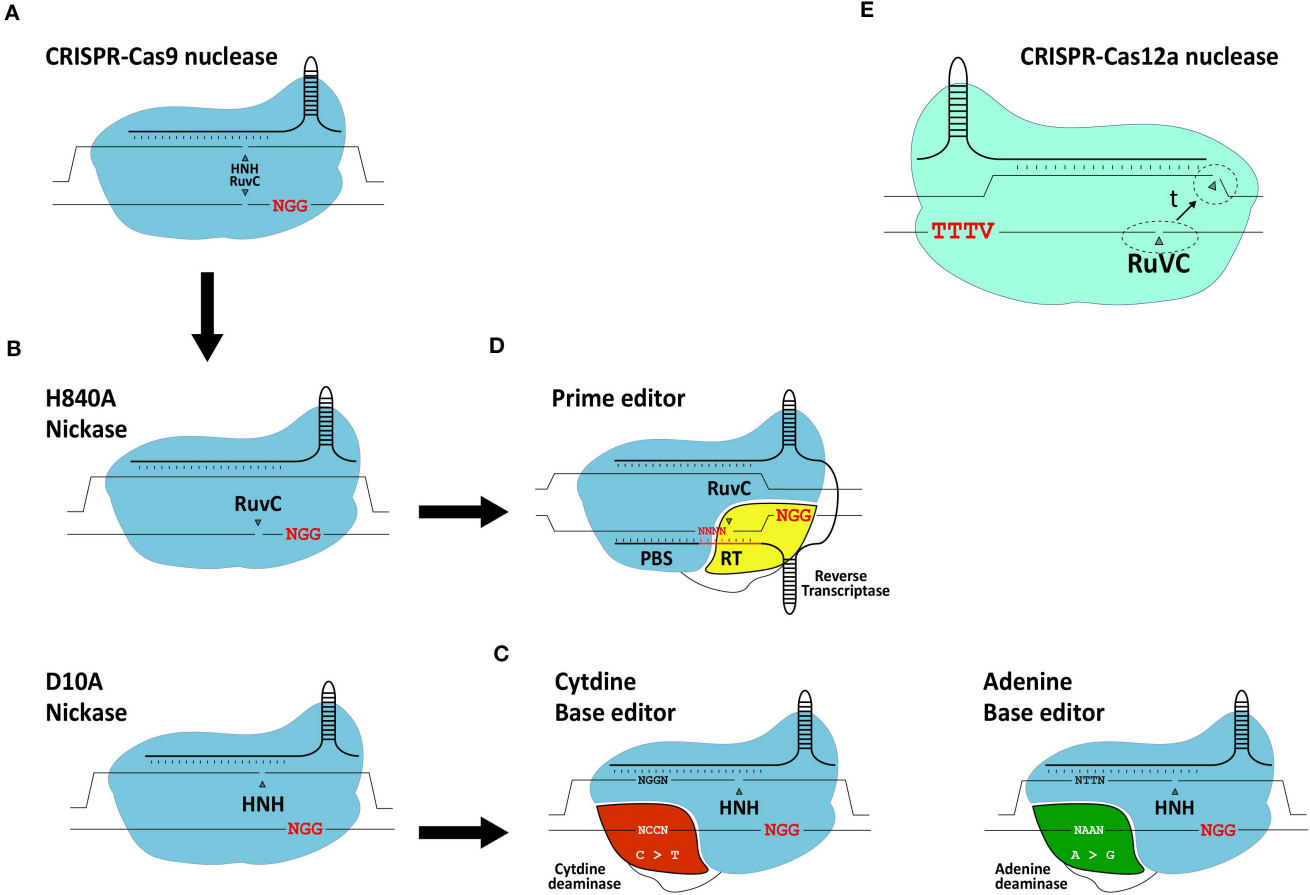
Schematic representation of gene editing tools based on CRISPR systems. **(A)** RGN based on the prototypic *S. pyogenes* CRISPR-Cas9 system. **(B)** RGNs containing the sequence- and strand-specific nuclease (nickase) Cas9^H840A^ or Cas9^D10A^. **(C)** Base editors. The basic components of base editors consist of a nickase (typically *S. pyogenes* Cas9^D10A^) fused to a cytidine deaminase or engineered adenine deaminase. **(D)** Prime editors. The basic components of prime editors consist of a nickase (typically *S. pyogenes* Cas9^*H*840*A*^) linked to an engineered reverse transcriptase. **(E)** RGN based on a CRISPR-Cas12a system. Cas12a has a single RuvC-like domain responsible for staggered DNA end formation from the sequential cleavage of both target site strands (dashed circles: location of cleavage; t: time). Red letters; protospacer adjacent motifs.

Shortly after the introduction of TALENs, a genome editing platform derived from prokaryotic class 2 type II CRISPR-Cas9 adaptive immune systems emerged (Cho et al., 2013; Cong et al., 2013; Jinek et al., 2013; Mali et al., 2013) (Figures 4, 5). This platform was built on plenty of fundamental insights culminating on the finding that Cas9 proteins from *Streptococcus thermophilus* and *Streptococcus pyogenes* are in fact RNA-guided site-specific endonucleases (Gasiunas et al., 2012; Jinek et al., 2012). Hence, as RGNs rely on RNA-DNA hybridizations for target DNA cleavage, they have a protein engineering-free mode of construction making them more easily customizable than ZFNs or TALENs (Doudna and Charpentier, 2014; Maggio and Gonçalves, 2015; Chandrasegaran and Carroll, 2016).

The adaptation of native RGNs into engineered RGNs designed to work in mammalian cells consisted of (i) assembling single-guide RNAs (gRNAs) by fusing sequence-tailored CRISPR RNAs (crRNAs) to a common scaffolding *trans*-activating CRISPR RNA (tracrRNA), (ii) codon-optimization of the Cas9 open reading frame; and (iii) addition of nuclear localization signals to the Cas9 protein (Cho et al., 2013; Cong et al., 2013; Jinek et al., 2013; Mali et al., 2013; Maggio et al., 2020). Once in target cells, Cas9:gRNA ribonucleoprotein complexes scan the genome for protospacer-adjacent motifs (PAMs), that read NGG in the case of *S. pyogenes* RGNs. This short DNA motif is recognized by the PAM-interacting domain (PID) of Cas9 (Anders et al., 2014). If next to the PAM lies a typically 18-21 nucleotide-long sequence (protospacer) complementary to the 5'-end of the crRNA (spacer), gRNA:DNA hybridization ensues leading to the activation of the two Cas9 nuclease domains (i.e., HNH and RuvC-like). The subsequent cleavage of the double-stranded DNA substrate occurs within the protospacer sequence typically three base-pairs away from the PAM.

Similarly to ZFNs and TALENs, RGNs can induce DSBs at off-target sequences (Cradick et al., 2013; Fu et al., 2013; Hsu et al., 2013; Frock et al., 2015; Chen et al., 2020). Therefore, to minimize deleterious effects caused by off-target DNA cleavage, considerable efforts are ongoing devoted to improve the precision of gene editing tools and strategies such as, by developing high-fidelity Cas9 nucleases and developing Cas9 nickase-based approaches which do not rely on the catalytic induction of DSBs. These precise gene editing technologies are briefly reviewed next.

#### Precision Gene Editing Based on High-Fidelity Nucleases, Nickases, Base Editors and Prime Editors

The generation and isolation of Cas9 variants with various point mutations through rational protein design and directed evolution approaches, respectively, have led to an array of high-fidelity Cas9 nucleases that next to greatly reduced off-target activities retain, for the most part, on-target efficiencies (Kim et al., 2019). Moreover, RGNs containing Cas9 nickases (Doudna and Charpentier, 2014) (Figure 5), developed through the disruption of one of the two aforementioned catalytic domains of the Cas9 nuclease, show interesting safety enhancements owing to the fact that the single-stranded DNA breaks (nicks) that they generate are intrinsically less disruptive than DSBs. Indeed, in the context of HDR-based gene editing experiments, researchers have found that coordinated nicking of target and donor DNA by Cas9 nickases can yield high gene knock-in frequencies while minimizing the characteristic by-products of Cas9 nucleases, i.e., NHEJ-derived indels at target and off-target sequences (Chen et al., 2017, 2020; Nakajima et al., 2018; Hyodo et al., 2020).

With the arrival of base editors, it is also now possible to introduce specific point mutations in living cells without the necessity for inducing DSBs or delivering donor DNA templates (Komor et al., 2016; Gaudelli et al., 2017) (Figure 5). Base editors consist of a Cas9 nickase (i.e., Cas9^D10A^) covalently linked to either a cytidine or adenine deaminase capable of inducing C → T or A → G transitions, respectively (Komor et al., 2016; Gaudelli et al., 2017). Typically, base editors induce these transitions within a 4-base pair (bp) base editing window. Similar to the growing number of nucleases derived from CRISPR systems evolved in different prokaryotes (Chen and Gonçalves, 2018), base editors are continuously being optimized to, for example, expand their PAM-recognition capabilities and reduce their off-target activities at both the genomic and transcriptomic levels (Koblan et al., 2018; Grunewald et al., 2019; Park and Beal, 2019; Zuo et al., 2019; Anzalone et al., 2020).

The most recent addition to the DSB-free gene editing toolkit independent of donor DNA delivery comes in the form of prime editors (Anzalone et al., 2019) (Figure 5). Although prime editing is a recent technique, so far mostly investigated in cell lines, it shows substantial potential including for the treatment of genetic disorders. In comparison with base editing, prime editing offers a broader range of targeted genomic edits in that it permits installing not only transitions but also transversions and small indels. Prime editors consist of a Cas9 nickase (i.e., Cas9^H840A^) covalently linked to an engineered Molony murine leukemia virus reverse transcriptase (RT) optimized owing to five point mutations enhancing its stability and processivity (Anzalone et al., 2019). Besides a Cas9^H840A^::RT complex, prime editing requires a 3'-end extended gRNA, named prime editor gRNA (pegRNA), that simultaneously provides a primer and a template for the RT. Specifically, the pegRNA consists of a conventional sequence-tailored gRNA linked to a primer binding site (PBS) and a RT template encoding the edit of interest. Via the PAM-interacting domain of the Cas9 nickase, the prime editor recognizes the PAM and, after hybridization between the crRNA portion of the pegRNA and the target sequence, the PAM-containing DNA strand is nicked. The resulting single-stranded genomic DNA anneals to the PBS of the pegRNA providing a primer for RT-mediated cDNA synthesis. The resulting DNA copy containing the edit eventually hybridizes to the complementary chromosomal target sequence with non-hybridizing DNA flap removal leading to edit installation at the target site presumably following DNA replication or mismatch repair (Anzalone et al., 2019).

#### CRISPR-Cas12a

The Cas12a nuclease, formally known as Cpf1, belongs to the class 2 type V CRISPR system found in, amongst other bacterial species, *Francisella novicida* (Makarova et al., 2020). In contrast to the *S. pyogenes* Cas9 nuclease, Cas12a from *F. novicida* (i) recognizes a T-rich PAM (i.e., TTTV), (ii) requires a crRNA and no tracrRNA and (iii) seems to have a single RuvC-like domain mediating the staggered cleavage of double-stranded DNA strands with 4-5 nt overhangs distal from the PAM (Zetsche et al., 2015) (Figure 5E). Optimization of the Cas12a nuclease is ongoing, mainly focusing on broadening their PAM recognition sites and further improving their intrinsically high target site specificities (Kleinstiver et al., 2016, 2019; Gao et al., 2017; Tóth et al., 2020).

### Genome Editing Strategies for the Treatment of Hemoglobinopathies

The tailoring of genome editing strategies for the genetic correction of hemoglobinopathies is progressing rapidly. These strategies can be divided in two main categories: (i) *HBB* mutation-independent; and (ii) *HBB* mutation-specific.

The mutation-independent approaches encompass the majority of these gene editing efforts in part owing to their compatibility with treating most patients regardless of their genotypes. Generically, mutation-independent strategies depend on the de-repression of HbF synthesis through the disruption of molecular mechanisms underlying ɤ-globin gene repression by, for instance, knocking-out repressor protein binding sites or the repressor genes themselves (Wienert et al., 2018; Demirci et al., 2020). Alternatively, instead of activating HbF synthesis, to complement the lack of β-globin, HDR-mediated gene editing is being exploited to directly correct the *HBB* gene itself via the introduction of programmable nucleases and donor DNA templates into HSCs (Dever et al., 2016; Antony et al., 2018; Pattabhi et al., 2019). The mutation-specific strategies are so far mostly based on targeted DSB formation for ablating aberrant splicing sites causing β-thalassemia (Patsali et al., 2019a). Although most gene editing efforts are directed toward the correction of β-hemoglobinopathies, i.e., β-thalassemia major and SCD, there are also studies focusing on the correction of α-thalassemia (Chang and Bouhassira, 2012; Yingjun et al., 2019).

#### *HBB* Mutation-Independent Genome Editing Strategies

**Figure 6:**
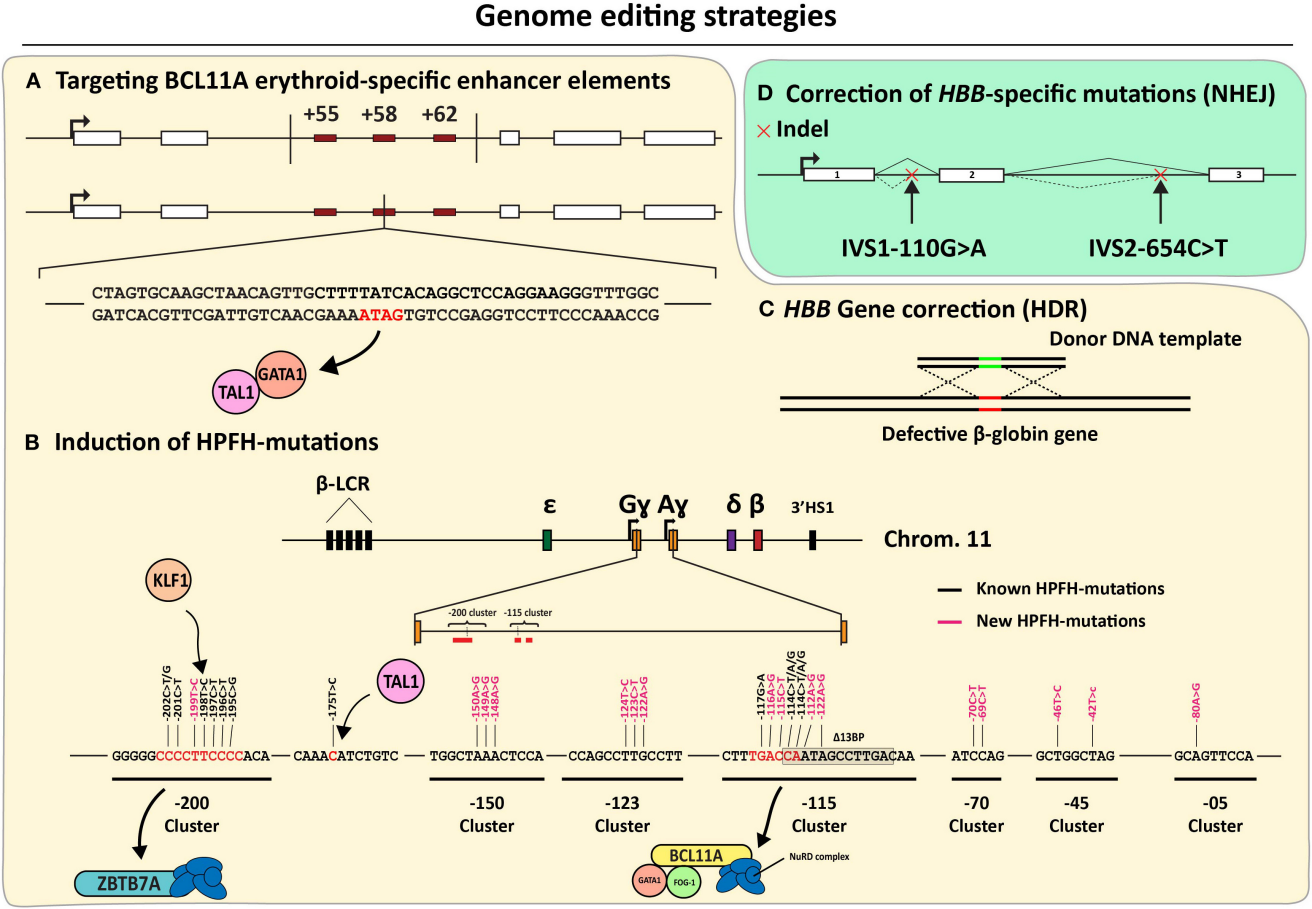
An overview of the genome editing strategies directed at correcting β-hemoglobinopathies. **(A–C)** Mutation-independent gene editing strategies. **(A)** Disruption of erythroid-specific *BCL11A* expression through the disablement of enhancer elements based on, targeted deletions or GATA1-binding site disruption. **(B)** Introduction of HPFH or HPFH-like mutations near the *HBG* transcriptional start sites. **(C)** HDR-dependent *HBB* correction. **(D)** Mutation-specific NHEJ-dependent strategies targeting aberrant splice motifs for the correction of β-thalassemia.

As discussed previously, insights into the fetal-to-adult hemoglobin switch continue being amassed in part owing to their importance for the development of mutation-independent gene complementation strategies for treating β-hemoglobinopathies, the world's most common group of monogenic disorders (Sankaran and Orkin, 2013; Vinjamur et al., 2018; Wienert et al., 2018). During the fetal-to-adult hemoglobin switch, ɤ-globin expression is repressed by molecular mechanisms involving key transcription factors, such as, BCL11A, LRF/ZBTB7A, SOX6, and KLF1 (Sankaran et al., 2008; Masuda et al., 2016; Li et al., 2017). Indeed, initial genome-wide association studies (GWAS) focused on identifying loci involved in fetal γ-globin repression, such as, *HBS1L-MYB* and *BCL11A* (Menzel et al., 2007; Uda et al., 2008). Furthermore, SNPs found in the β-globin locus of SCD patients with elevated HbF levels uncovered *cis*-acting elements controlling fetal γ-globin repression (Lettre et al., 2008). Since then, the regulation of *HBS1L-MYB* and *BCL11A*, underwent extensive investigations to confirm and further elucidate the nodal role of these genes in the control of fetal γ-globin gene expression (Lettre et al., 2008; Fanis et al., 2014). For instance, the BCL11A-mediated control of γ-globin gene expression was confirmed through shRNA-mediated knockdown experiments (Sankaran et al., 2008). Importantly, besides its role in the erythrocytic lineage, the DNA-binding protein BCL11A is also involved in other key hematopoietic processes, such as, in the control of HSC differentiation and quiescence as well as lymphoid development (Liu et al., 2003; Tsang et al., 2015; Luc et al., 2016). Thus, to prevent systemic ablation of BCL11A in all hematopoietic lineages, it is important to restrict BCL11A-disrupting genetic interventions to the erythrocytic compartment. Crucially, three DNase I HS sites located in the intron 2 of *BCL11A* were identified and confirmed to represent erythroid-specific *BCL11A* enhancers (Bauer et al., 2013) (Figure 6). The first gene editing experiments targeting these erythroid-specific enhancer elements were done by using CRISPR-Cas9-based RGNs to dissect their individual and combined roles through targeted DNA deletions (Canver et al., 2015) (Figure 6). This study, besides confirming the importance of the three *BCL11A* enhancer elements in repressing ɤ-globin expression, has also identified the particularly significant contribution of the so-called +58 enhancer element in this process (Canver et al., 2015). The reason for this heightened contribution in ɤ-globin repression was hypothesized to result from the binding of transcription-activating GATA1/TAL1 complexes to a GATA1 binding site present within the +58 enhancer (Bauer et al., 2013; Bauer and Orkin, 2015) (Figure 6A). Recent gene editing experiments, using both ZFNs and CRISPR-Cas9-based RGNs, targeting this specific GATA1 binding site confirmed its crucial function in controlling *BCL11A* expression within the erythrocytic lineage. As a result, these experiments further support increasing fetal γ-globin levels through NHEJ-mediated disruption of the GATA1/TAL1-binding sequence in the +58 enhancer (Chang K. H. et al., 2017; Psatha et al., 2018; Wu et al., 2019) (Figure 6A). Moreover, a more exquisite ablation of the GATA1-binding site in the *BCL11A* +58 erythroid enhancer was recently achieved by using base editing (Zeng et al., 2020). In this study, high editing efficiencies led to the up-regulation of γ-globin synthesis in RBCs differentiated from SCD patient-derived CD34^+^ cells. This data indicates that base editing is a promising approach for treating SCD and β-thalassemia (Zeng et al., 2020). Although the relevance of BCL11A in fetal γ-globin repression has been clearly established through the abovementioned *BCL11A*-targeting genetic studies, additional and potentially complementary gene editing strategies are evolving. Amongst these are those based on targeting the binding sites of repressor proteins located within the regulatory regions of the γ-globin-encoding *HBG* genes themselves. Inspired by naturally occurring HPFH-conferring mutations, two regions upstream of the *HBG* promoter sequences were identified as LRF/ZBTB7A and BCL11A binding sites, i.e.,−200 bp and −115 bp distal from the *HBG* transcription start sites (Martyn et al., 2018) (Figure 6B: −115 and −200 clusters). A similar contemporary study on the interactions between BCL11A and regulatory DNA near the *HBG* promoters revealed a distal TGACCA motif that proved to be essential for BCL11A binding (Liu et al., 2018). The functional importance of LRF/ZBTB7A and BCL11A binding sites on the control of *HBG* expression, was further confirmed through their targeted disruption by CRISPR-Cas9-based RGNs (Liu et al., 2018; Martyn et al., 2018).

Based on these findings, and the knowledge on naturally occurring mutations conferring HPFH phenotypes, gene editing strategies are being developed aiming at disrupting or removing these specific *HBG* repressor binding sites and mimicking other HPFH-conferring mutations (Wienert et al., 2015; Traxler et al., 2016; Ye et al., 2016; Antoniani et al., 2018; Humbert et al., 2019; Lux et al., 2019; Métais et al., 2019; Wang et al., 2020; Weber et al., 2020) (Figure 6B). Moreover, several *ex vivo* genetic therapy experiments in animal models using gene editing tools designed for the de-repression of ɤ-globin expression achieved promising results without compromising the functionality of the transplanted stem cells (Humbert et al., 2019; Lux et al., 2019; Métais et al., 2019; Weber et al., 2020). Interestingly, by using CRISPR-Cas12a-based RGNs, researchers were able to mimic an HPFH-causing 13-bp deletion within the CCAAT-box of the *HBG* promoter in CD34^+^ cells (De Dreuzy et al., 2019). In these experiments, high editing frequencies were measured (80–90%) resulting in a 40% increase in HbF levels. Importantly, cell engraftment followed by long-term polyclonal multilineage repopulation was achieved upon transplantation of the treated CD34^+^ cell populations into NBSGW mice (De Dreuzy et al., 2019).

Currently, two strategies targeting the GATA1 binding site in the *BCL11A* +58 erythroid-specific enhancer have entered clinical trials for both β-thalassemia and SCD (Table 2). The *ex vivo* genetic therapy strategy utilizing the CRISPR-Cas9-based RGN platform to treat SCD (clinical trial NCT03745287), so far resulted in 6 patients treated of which 3 patients remained vaso-occlusive crisis free (VOC-free) with fetal hemoglobin levels up to 48% (Table 2). When applied in β-thalassemia patients (clinical trial NCT03655678), this strategy led to elevated HbF levels (ranging from 40.9% to 97.7%) and, importantly, blood transfusion independency for 7 out of 13 patients infused with CTX001 (Table 2). Another strategy makes use of ZFNs to disrupt the GATA1 binding site of the +58 erythroid-specific enhancer of *BCL11A* in cells from SCD and β-thalassemia patients (clinical trials NCT03653247 and NCT03432364, respectively) (Table 2). Other clinical trials involving the *ex vivo* gene editing and subsequent transplantation of autologous CD34^+^ cells for treating patients with SCD and β-thalassemia, are ongoing (Table 2).

**Table 2:** Genetic therapies in development based on gene editing.

**Phase**	**Product name**	**Clinical trial**	**Target cell**	**Delivery**	**Nuclease**	**Target gene & effect**	**Disorder**	**Status**	**Sponsor**	**Results**
I/II	ST-400	NCT03432364	CD34^+^	Electroporated mRNA	ZFN	Disruption of erythroid enhancer of *BCL11A* gene	TM	Active/Not recruiting	Sangamo	**Five patients (3/5 prelim. data): 1/3**−23% on-target indels 2.7 g/dl HbF (0.9 g/dl at baseline); **2/3**−73% on-target indels <1 g/dl HbF; **3/3**−54% on-target indels 2.8 g/dl HbF
I/II	BIVV003	NCT03653247	CD34^+^	Electroporated mRNA	ZFN	Disruption of erythroid enhancer of *BCL11A* gene	SCD	Recruiting	Bioverativ (Sanofi)	No results published yet
I/II	CTX001 (CLIMB-111)	NCT03655678	CD34^+^	Electroporated RNP	CRISPR-Cas9	Disruption of erythroid enhancer of *BCL11A* gene	TM	Recruiting	Vertex pharmaceuticals inc.	13 patients treated. TM: 7/13 TI with 3-18 months of follow-up. Total Hb from 9.7 - 14.1 g/dL and fetal Hb levels from 40.9 - 97.7%
I/II	CTX001 (CLIMB-121)	NCT03745287	CD34^+^	Electroporated RNP	CRISPR-Cas9	Disruption of erythroid enhancer of *BCL11A* gene	SCD	Recruiting	Vertex pharmaceuticals inc.	6 patients treated. VOC-free: 3/3; 3-15 months after CTX001 infusion. Total Hb from 11.5 - 13.2 g/dL and fetal Hb levels from 31.3 - 48%.
I/II	-	NCT04211480	CD34^+^	Electroporated RNP	CRISPR-Cas9	*BCL11A* binding site disruption *HBG* promoter	TM	Recruiting	Shanghai Bioray Laboratory Inc.	**2 patients**: 1 MPT HbF levels increased to 76 and 97 g/L HbF (total Hb 129 and 115 g/L, resp.) TI at 75 DPT
Long-term follow-up	CTX001	NCT04208529	CD34^+^	Electroporated RNP	CRISPR-Cas9	Disruption of erythroid enhancer of *BCL11A* gene	TM & SCD	-	Vertex pharmaceuticals inc.	-

*TM, beta thalassemia major; TI, Transfusion independent; DPT, Days post-treatment; MPT, Months post-treatment; VOC, Vaso-occlusive crises*.

Although the current focus is on installing mutations known to confer a naturally occurring HPFH phenotype, eight *de novo*-created mutations yielding a HPFH-like phenotype have recently been presented in yet to be peer reviewed data (Ravi et al., 2020). These mutations have been identified by the systematic induction of point mutations by base editors at *HBG* distal regulatory regions or at sequences near the *HBG* transcription start site (Figure 6B). These recent findings broaden the range of candidate *HBG* target sites and in doing so, increase the options for therapeutic gene editing based on the de-repression of ɤ-globin protein synthesis.

Besides creating a HPFH-like phenotype through the installation of NHEJ-derived indels and point mutations at *BCL11A* and *HBG* alleles through the delivery of programmable nucleases and base editors, respectively, other gene editing strategies under investigation comprise instead HDR-mediated *HBB* correction (Figure 6C). However, achieving HDR-mediated gene editing in *bona fide* HSCs is challenging due to their mostly quiescent nature and poor amenability to transfection and transduction methods needed to deliver the necessary gene editing tools (i.e., donor DNA templates and RGNs). Exposing HSCs *ex vivo* to small-molecule drugs and growth factors favoring survival and limited cell cycle entry can improve gene editing frequencies (Genovese et al., 2014) but not without, at least in part, impacting their basic properties of life-long self-renewal and multi-lineage differentiation capacities.

Despite this, there are studies providing proof-of-principles for the targeted integration of exogenous DNA sequences in HSCs by using ZFNs, TALENs and RGNs (DeWitt et al., 2016; Antony et al., 2018; Pattabhi et al., 2019). Of notice, direct comparison of these three programmable nuclease platforms showed particularly high NHEJ-derived indel frequencies when using the CRISPR-Cas9-based RGN platform (Antony et al., 2018). Furthermore, Pattabhi and coworkers compared HDR-mediated *HBB* editing in HSCs using AAV9 vs. single-stranded oligodeoxyribonucleotide (ssODN) donors (Pattabhi et al., 2019). Moreover, Shinn and colleagues have been able to demonstrate that controlled proliferation and quiescence of HSCs can yield up to a 6-fold increase in HDR/NHEJ ratios in human CD34^+^ cells *in vitro* and *in vivo* (Shin et al., 2020). Although various research efforts have been improving HDR efficiencies in HSCs, additional tools and/or protocol modifications are likely to be required in order to achieve gene editing frequencies clinically meaningful to the sustained rescue of SCD and β-thalassemia phenotypes.

Another mutation-independent gene editing strategy, albeit less pursued, consists of down-regulating α-globin expression and, therefore, establishing a more balanced proportion amongst the hemoglobin chains. Amongst the first *HBA1*-targeting approaches were those based on RNA interference encompassing the delivery of small interfering RNA (siRNA) or shRNA molecules into murine β-thalassemic primary erythrocytes (Voon et al., 2008), β-thalassemia heterozygous single-cell mouse embryos (Xie et al., 2007) and a β-thalassemia mouse model after tail vein injection (Xie et al., 2011; Mettananda et al., 2015). More recently, a study based on the delivery of RGN multiplexes designed for the targeted deletion of the aforementioned MCS-R2 regulatory element resulted in a reduced α-globin synthesis with a corresponding reduction in hemoglobin chain imbalances in primary human HSCs (Mettananda et al., 2017).

#### *HBB* Mutation-Specific Genome Editing Strategies

Concerning the *HBB* mutation-specific gene editing strategies for hemoglobinopathies, these have hitherto mostly targeted mutations that create cryptic splice sites that, via aberrant splicing and ensuing coding sequence frameshifts, cause β-thalassemia (Patsali et al., 2019b; Xu et al., 2019). By relying on the installation of indels after NHEJ-mediated repair of DSBs induced by Cas9 or Cas12a nucleases, higher *HBB* correction efficiencies are achieved than those involving HDR-mediated gene editing. Since these type of mutations (e.g., IVS1-110G>A and IVS2-654C>T) are particularly common (Kountouris et al., 2014), disruption of aberrant regulatory elements (DARE) approaches might become highly relevant for the correction of the disease in the β-thalassemia patient population (Patsali et al., 2019a) (Figure 6D). However, it is worth mentioning that the high sequence identity between *HBB* and other genes in the β-globin locus cluster heightens the risk for adverse events stemming from intra- and inter-chromosomal rearrangements in the form of, for instance, large deletions and translocations, respectively (Long et al., 2018). Hence, concerning this issue, it should be valuable investigating the efficiency and accuracy of DARE via DSB-free base editing and prime editing as alternative approaches to programmable nuclease-induced indel formation.

## Conclusions and Prospects

The development of genetic therapies for treating hemoglobinopathies is progressing at a sustained pace. Gene therapy technologies based on LV-mediated *HBB* gene supplementation have in fact reached advanced clinical trial stages (Table 1). Although improved LV designs present reduced safety concerns associated with insertional oncogenesis (e.g., HIV-1 SIN constructs with miniaturized *HBB* enhancer/promoter elements), life-long monitoring for the emergence of potentially hazardous monoclonal expansions of HSC progenies, is warranted. Importantly, LV-based gene therapies are showing promising results in terms of achieving clinically relevant β-like globin expression levels in initial and ongoing clinical trials (Table 1) (Sii-Felice et al., 2020).

The gathering of fundamental insights on developmentally regulated ɤ-globin repression and chromatin looping mechanisms, constitute additional significant developments as they guide the search for novel genetic therapies (Krivega and Dean, 2016; Wienert et al., 2018; Brendel et al., 2020). Moreover, one should equally stress the crucial contribution of clinical genetics to the unraveling of such fundamental mechanisms of hemoglobin biology. Indeed, by learning from and leveraging upon distinct natural mutations causing pathology or phenotype amelioration, it is now possible to pursue *HBB* mutation-independent and *HBB* mutation-specific genetic therapies. Indeed, gene editing platforms with increasing accuracy are offering the prospect for modifying genomic sequences underlying severe hemoglobinopathies through either mutation-dependent or independent strategies. Promising results from pre-clinical models and early-stage clinical trials point to a role for gene editing in the treatment of hemoglobinopathies (Table 2). Despite these developments, further improvements are clearly in demand to establish genome editing as a broadly applicable and safe therapeutic option for hemoglobinopathies. The need for high corrective-gene expression levels and high frequencies of gene edited cells means that improving gene editing tools must go hand-in-hand with implementing systems for their delivery into *bona fide* HSCs.

In this context, episomal (i.e., non-integrating) viral vectors, such as adenoviral (AdV) vectors and adeno-associated viral (AAV) vectors, are promising agents for introducing gene editing components into HSCs, in particular, certain capsid pseudo-typed variants (Chen and Gonçalves, 2016; Li and Lieber, 2019; Li C. et al., 2020; Tasca et al., 2020; Yang et al., 2020). For instance, AAV serotype 6 (AAV6) seems to be particularly effective in transducing HSCs when compared to other AAV serotypes, making it a valuable platform to test, amongst others, HDR-mediated *HBB* gene correction strategies (Pattabhi et al., 2019; Yang et al., 2020). It is also possible that, in addition to their efficient HSC transduction, the peculiar structure of AAV vector genomes, consisting of single-stranded DNA ended by palindromic inverted terminal repeats, contributes to donor-target DNA recombination (Holkers et al., 2012). On the other hand, recent experiments indicate that AAV vector genomes trigger a p53-dependent DNA damage response in HSPCs and, through non-homologous recombination processes, integrate at significant rates at RGN target sites in murine tissues (Hanlon et al., 2019; Nelson et al., 2019; Schiroli et al., 2019). The latter data are consistent with earlier results disclosing that a measurable fraction of AAV donor DNA becomes “captured” at ZFN-induced DSBs in murine livers (Li et al., 2011; Anguela et al., 2013). These events might be most problematic in cases where AAV vector genomes encode programmable nucleases as they directly raise issues concerning the permanency of these tools in transduced cells.

Collectively, these findings stress the need to (i) closely monitoring the impact and precision of gene repair procedures in target cells regardless of their type and replication status (Maggio and Gonçalves, 2015); and (ii) expand the range of delivery agents that, like AAVs, are devoid of viral genes but that, in contrast to these vectors, permit transferring recombinant DNA larger than ~4.7 kb; which is the packaging capacity of AAV capsids. Concerning the latter aspect, high-capacity AdV vectors endowed with the cell tropism of species B adenoviruses (e.g., serotypes 35 and 50) are valuable candidates owing to their efficient transduction of HSCs and high genetic payload (i.e., up to 36 kb) (Li and Lieber, 2019; Tasca et al., 2020).

Equally regarding the ultimate performance of gene editing interventions, it is worth mentioning that various types of stem and progenitor cells, including HSPCs, are particularly susceptible to p53-dependent cell cycle arrest and apoptosis, even when subjected to a limited number of targeted DSBs (Haapaniemi et al., 2018; Ihry et al., 2018; Schiroli et al., 2019). Moreover, besides triggering intended gene editing outcomes, targeted DSBs can negatively impact the genotype and phenotype of gene edited cells by installing potentially hazardous allelic and non-allelic chromosomal rearrangements and decreasing cell fitness, respectively (Frock et al., 2015; Kosicki et al., 2018; Chen et al., 2020).

Hence, looking ahead, besides seeking to enhance absolute gene editing efficiencies, an equally important priority will be continuing to improve the safety profile of gene editing procedures as a whole. To this end, macromolecular enzymatic complexes that bring about targeted and precise genomic modifications without catalytic induction of DSBs might become particularly valuable and include, nicking RGNs and their derivative base and prime editor proteins as well as engineered or molecularly evolved site-specific recombinases and CRISPR-based transposases and recombinases (Komor et al., 2016; Chen et al., 2017; Gaudelli et al., 2017; Nakajima et al., 2018; Anzalone et al., 2019, 2020; Klompe et al., 2019; Strecker et al., 2019; Hyodo et al., 2020).

In conclusion, knowledge from hemoglobin biology and clinical genetics studies, together with the herein covered rapid expansion of gene editing techniques, are accelerating the development of genetic therapies for treating hemoglobinopathies. These advances are in turn expected to capitalize and build upon gene transfer and stem cell technologies underlying *ex vivo* transduction and autologous transplantation of HSCs into afflicted patients. This being said, in addition to regulatory requirements, robust and affordable GMP-grade platforms for up-scaling and downstream processing of AMTPs will be crucial before genetic therapies for hemoglobinopathies become broadly available to those in need (Staal et al., 2019).

## Author Contributions

HZ wrote the manuscript with the contributions from the other authors. All authors have reviewed and edited the work.

## Conflict of Interest

The authors declare that the research was conducted in the absence of any commercial or financial relationships that could be construed as a potential conflict of interest.
